# Effect of irrigation fluid temperature on body temperature during arthroscopic elbow surgery in dogs

**Published:** 2013-10-19

**Authors:** K.R. Thompson, P.D. MacFarlane

**Affiliations:** *Small Animal Teaching Hospital, University of Liverpool, Leahurst Campus, Chester High Road, Neston, Cheshire CH64 7TE, UK*

**Keywords:** Arthroscopy, Hypothermia, Irrigation fluid temperature, Thermoregulation

## Abstract

This prospective randomised clinical trial evaluated the effect of warmed irrigation fluid on body temperature in anaesthetised dogs undergoing arthroscopic elbow surgery. Nineteen dogs undergoing elbow arthroscopy were included in the study and were randomly allocated to one of two groups. Group RT received irrigation fluid at room temperature (RT) while dogs in group W received warmed (W) irrigation fluid (36°C). A standardised patient management and anaesthetic protocol was used and body temperature was measured at four time points; (T1) pre-anaesthetic examination, (T2) arrival into theatre, (T3) end of surgery and (T4) arrival into recovery. There was no significant difference in body temperature at any time point between the groups. The mean overall decrease in body temperature between pre-anaesthetic examination (T1) and return to the recovery suite (T4) was significant in both groups, with a fall of 1.06±0.58°C (p<0.001) in group RT and 1.53±0.76°C (p<0.001) group W. There was no significant difference between the groups. At the end of surgery (T3) 4/19 (21.1%) of dogs were hypothermic (<37°C). The addition of warmed irrigation fluids to a temperature management protocol in dogs undergoing elbow arthroscopy during general anaesthesia did not lead to decreased temperature losses.

## Introduction

Hypothermia during and after anaesthesia is common in dogs, with up to 83.5% of dogs developing hypothermia in a recent large study (Redondo *et al.*, 2012). In human anaesthesia hypothermia is also a common problem (Vaughan *et al.*, 1981), and even mild hypothermia [34.0-36.5°C (Kirkbride and Buggy, 2003)] is associated with increased rates of wound infection (Kurz *et al.*, 1996; Melling *et al.*, 2001), adverse cardiac events (Frank *et al.*, 1997) and mortality in man (Karalapillai *et al.*, 2009).

There is limited evidence to document similar adverse events associated with hypothermia in dogs and a retrospective veterinary study showed no increase in wound infection rates in cats and dogs with mild perioperative hypothermia (Beal *et al.*, 2000). However, the link between hypothermia and delayed recovery from anaesthesia is well established (Lenhardt *et al.*, 1997; Sinclair and Faleiro, 2006).

Increasing emphasis is being placed on prevention of peri-operative hypothermia in both man (NICE, 2008) and dog (Dhupa, 1995; Oncken *et al.*, 2001; Armstrong *et al.*, 2005). In both dogs and humans, warming fluids used for irrigation of body cavities has generally been associated with a protective effect against peri-operative hypothermia (Pit *et al.*, 1996; Nawrocki *et al*., 2005; Okeke, 2007) and this practice has been incorporated into UK national guidelines on peri-operative temperature management in man as a simple, low cost intervention (NICE, 2008).

The use of warmed arthroscopic irrigation fluids in human patients undergoing arthroscopic shoulder surgery has been associated with a smaller reduction in core body temperature compared to the use of fluids at room temperature with differences in maximum temperature drop as large as 1.34°C being reported between groups (Board and Srinivasan, 2008; Kim *et al.*, 2009). However, Kelly *et al*. (2000) found no significant difference in mean percentage temperature decrease between patient groups treated with warmed and room temperature irrigation fluids undergoing knee arthroscopy. The use of warmed irrigation fluids for arthroscopy has not been previously investigated in dogs. It was hypothesised that the use of pre-warmed arthroscopic irrigation fluid would result in less heat loss during the surgical period compared to the use of room temperature irrigation fluid.

## Materials and Methods

Nineteen dogs admitted for arthroscopic elbow surgery under general anaesthesia were recruited for the study. All patients presenting at the hospital for elbow arthroscopy were considered for inclusion in this study. Patients were excluded from the study if they met ASA patient category III-V or weighed less than 10kg. Informed owner consent was obtained prior to inclusion of any patient in the study.

The study was reviewed by the University of Liverpool Research Ethics Committee and given approval (VREC01).

A clinically significant difference in heat loss between groups was considered to be one degree centigrade and a power analysis based on this suggested that in order to achieve an alpha of 0.05% with power of 85% 8 dogs would be required in each group. In order to allow for potential data loss it was planned to recruit 10 dogs to each group (20 in total). Due to a change of surgical policy in the hospital the number of arthroscopies performed decreased dramatically during the course of the study and it proved possible to recruit only 19 dogs with 10 in group RT and 9 in group W.

### Anaesthetic protocol

All dogs received a premedication combination of acepromazine (0.03mg kg^-1^ intramuscularly) (ACP injection 2mg mL^-1^; Novartis Animal Health, UK) and buprenorphine (0.02mg kg^-1^ intramuscularly) (Vetergesic 0.3mg mL^-1^ solution for injection for cats and dogs; Alstoe Animal Health, UK) administered approximately 45 minutes prior to induction of anaesthesia. Anaesthesia was induced with alfaxalone (Alfaxan 10mg mL^-1^ solution for injection for cats and dogs; Vétoquinol, UK) administered intravenously to effect.

Robenocoxib (2mg kg^-1^ subcutaneously) (Onsior 20mg mL^-1^ solution for injection for cats and dogs; Novartis Animal Health, UK) or an alternate NSAID [(meloxicam 0.2mg kg^-1^ intravenously; Metacam 5mg/ml solution for injection in cats and dogs; Boehringer Ingelheim, UK) or (carprofen 4mg kg^-1^ intravenously; Rimadyl 50mg/ml solution for injection in cats and dogs; Zoetis Ltd, UK)] depending on existence of preoperative NSAID regimen and amoxicillin clavulanate (20mg kg^-1^ intravenously) (Augmentin 100mg mL^-1^; GlaxoSmithKline, UK) were administered after induction of general anaesthesia.

Anaesthesia was maintained with isoflurane (IsoFlo; Abbott laboratories Ltd, UK) delivered in 100% oxygen via a small animal circle breathing system. Intra-operative intravenous fluid therapy consisted of room temperature Hartmann’s solution (5ml kg^-1^ hr^-1^) (Aqupharm No11; AnimalCare, UK).

If additional fluids were required then boluses of 10ml kg^-1^ Hartmann’s solution were administered intravenously. If additional analgesia was required, fentanyl (2µg kg^-1^) (Fentanyl; Martindale Pharmaceuticals, UK) was administered intravenously.

All dogs had a heat and moisture exchanger (HME) (Hydro Therm II ®; Intersurgical, UK) placed between the endotracheal tube and breathing system after endotracheal intubation. Once moved into the operating theatre, dogs were placed on a thermostatically controlled blanket (Hot Dog^®^; Augustine Biomedical and Design, MN, USA) which was set to 37°C throughout the surgical duration.

At the end of surgery bupivacaine hydrochloride (1mg kg^-1^) (Marcaine 0.25% preservative free; Hospira, UK) or morphine (0.1mg kg^-1^) (Morphine Sulphate 10mg mL^-1^ Martindale Pharmaceuticals, UK) or a combination of both were administered intra-articularly.

### Experimental Protocol

Dogs were randomly allocated to each group using sealed randomised envelopes selected by the attending theatre nurse. The anaesthetist was blinded to the temperature of the fluids selected. Fluids for arthroscopy where stored either at room temperature (20-22ºC) or pre-warmed in a thermostatically controlled heating cabinet (36ºC) for a minimum of 24 hours.

A 10ml sample of saline was withdrawn from a 1 litre test bag of saline kept in the incubator to ensure agreement (+/- 0.5°C) with the incubator thermostat prior to the start of each case. A separate 1 litre test bag of saline was stored in the theatre suite from which 10ml samples were withdrawn and used to verify fluid equilibration with room temperature (20-22°C) and also agreement between rectal and oesophageal temperature probes prior to the start of each case.

Irrigation fluids were removed either from the heated cabinet or theatre suite fluid store and mounted in the arthroscopy tower immediately prior to the onset of joint irrigation by the attending theatre nurse. The fluid was delivered to the joint via 4.8m tubing at a variable rate. No insulation was applied to the irrigation fluid bags.

Under experimental conditions designed to mimic ambient temperature conditions during the study, a 2l bag of pre-warmed irrigation fluid (36.4°C) was mounted in the arthroscopy tower at room temperature (20.5°C) and fluid temperature at the point of outflow from the delivery tubing was measured over 60 minutes.

### Body Temperature Measurement

Body temperature at time points T2 and T3 was measured using an oesophageal temperature probe connected to a multi-parameter monitor (Datex-Ohmeda S/5^®^ Anaesthesia Monitor; GE Healthcare, UK) and positioned approximately at the level of the carina.

An assigned rectal temperature probe (Sure Temp^®^ Plus; Welch Allyn Ltd, UK) was used to measure temperature at T1 and T4 time points. Temperature measurement of a 10ml volume of room temperature saline was used to verify agreement between the oesophageal temperature probe and rectal probe prior to the start of each case. The rectal and oesophageal probes recorded identical temperatures on each occasion.

### Statistical analysis

The data was assessed for normality using graphical representation and the Anderson-Darling test. Baseline data including; age, weight, anaesthetic duration, surgical duration, theatre temperature and preparation time was analysed using a two sample *t*-test and data are presented as mean ± standard deviation.

A one-way ANOVA was used to test for differences between the two groups at each of the four time points and time periods. A one-way ANOVA was also performed to assess temperature change over time within each group. *Post-hoc* Bonferroni *p*-value adjustments were made for multiple pair-wise comparisons. P-values <0.05 were considered significant. All statistical analyses were performed using Minitab 16^®^ computer software (Minitab Ltd).

## Results

No statistically significant differences existed between the groups in baseline data (Table 1).

**Table 1:** Baseline data for nineteen dogs receiving room temperature (RT) or warmed (W) irrigation fluids presented as mean ± standard deviation.

Group	Theatre Room Temperature (°C)	Preparation Time (mins) (Pre-medication to induction)	Anaesthetic Duration (mins)	Surgery Duration (mins)	Age (years)	Weight (kg)
Room temperature fluid (RT) group	20.78±2.28	39.7±7.29	88.5±26.6	54.0±20.8	1.83±1.37	24.52 ±6.20
Warmed fluid (W) group	20.69±1.10	42.8±18.4	95.6±30.8	58.9±28.0	2.95 ±2.75	27.33 ±6.46
*P* value for the difference between groups.	0.91	0.65	0.60	0.68	0.29	0.35

There was no statistically significant difference in temperature between the groups at any time point (Table 2).

**Table 2:** Data (mean ± standard deviation °C) for temperature measurements at each time point for nineteen dogs receiving room temperature (RT) or warmed (W) irrigation fluids.

Variable	Group	T1	T2	T3	T4
Temperature (°C)	RT	38.45 ±0.48	37.52 ±0.42*	37.40±0.62*	37.39±0.64*
			*p*<0.001	*p*=0.001	*p*=0.001
	W	38.83 ±0.43	37.87 ±0.54*	37.36±0.61*	37.30 ±0.54*
			*p*=0.001	*p*<0.001	*p*<0.001
P value for difference between groups		0.087	0.132	0.877	0.747

* Value differs significantly (*p*<0.05) from baseline (T1) value.

Within each group, temperature recorded at T2, T3 and T4 differed significantly from T1 (Table 2).

Within each group temperature reduction over time was significant between T1 and T2 (Table 3). Temperature reduction over time was not found to be significant between T2-T3 and T3-T4 (Table 3).

**Table 3:** Data (mean ± standard deviation °C) for temperature reduction over time periods for nineteen dogs receiving room temperature (RT) or warmed (W) irrigation fluids.

Variable	Group	T1-T2	T2-T3	T3-T4	T1-T4
Temperature Fall (°C)	RT	0.93±0.57*	0.12±0.50	0.01±0.23	1.06±0.58*
		*p*=0.001	*p*=0.466	*p*=0.89	*p*<0.001
	W	0.97±0.68*	0.51 ±0.47	0.06±0.39	1.53±0.76*
		*p*=0.003	*p*=0.012	*p*=0.68	*p*<0.001
P value for difference between groups		0.94	0.1	0.76	0.195

* Significant difference (*p*<0.05) over the time period within the group

Overall temperature reduction (T1-T4) was significant in each group representing overall heat loss from the time of pre-anaesthetic examination to transfer into recovery (Table 3).

In group RT, a temperature reduction of 1.06±0.58 *(p*<0.001) was recorded and in group W, a temperature reduction of 1.53±0.76 (*p<*0.001) was recorded between T1-T4 (Table 3). Using pooled data, temperature reduction in the preparatory phase (T1-T2) was 0.979±0.139°C and 0.305±0.514°C during surgery (T2-T3). Using pooled data, mean preparation time was shorter than mean surgery time. At the end of surgery (T3) 4/19 (21.1%) of dogs were hypothermic (< 37°C).

Data recorded under experimental conditions to imitate fluid cooling reported that the pre-warmed (36.4°C) fluid temperature decreased by 0.6°C after passing through the delivery tubing. After 12 minutes of administration, the fluid temperature at the outflow point was 35.1°C and after 40 minutes the fluid temperature was 32.0°C. After 60mins, temperature of the irrigation fluid at the outflow point was 29.6°C. Mean arthroscopy duration in group W was 43.3±20.62 mins.

## Discussion

The results of the current study suggest that the use of pre-warmed irrigation fluid for elbow arthroscopic surgery in dogs does not confer a thermoregulatory advantage when additional heating methods are employed. This is the first such study in dogs, and the results agree with those of Kelly *et al*. (2000) in man. However, Board and Srinivasan (2008) and Kim *et al*. (2009) demonstrated a positive association between the use of warmed irrigation fluid and body temperature during shoulder arthroscopy in man. These studies involved the use of much higher volumes of irrigation fluid over a longer period of surgical time (8.8 litres over 84 minutes and 10.05 litres over 92.8 minutes respectively) compared to the present study where irrigation fluid volume was less than 2 litres and mean surgery time 57.5 minutes. It may be that during a longer surgical period there is more time for heat loss to occur, potentially magnifying the difference between the groups.

Kelly *et al*. (2000) failed to find a positive association between body temperature and irrigation fluid temperature during knee arthroscopy in man where the inclusion of spinal anaesthesia may have compromised thermoregulatory control (Leslie and Sessler, 1996) leading to further heat loss in these patients.

A further difference between the reported studies is that in at least one of the human papers which reported a positive effect of fluid temperature, efforts were made to maintain the temperature of the irrigation fluid once removed from the warming cabinet (Kim *et al.*, 2009). No such attempt was made in our study or the human study which did not detect a positive effect of warmed fluid and so the fluid delivered to the patient cooled as the procedure proceeded (Kelly *et al.*, 2000). Conditions designed to mimic the current study setting showed that pre-warmed irrigation fluid temperature cooled with time but remained above 31°C for the mean arthroscopy time in group W of 43.3 minutes.

Kelly *et al*. (2000) carried out similar testing and found a fluid temperature reduction of 2°C over their 20 minute typical infusion period, however their pre-warmed saline was heated to 40°C rather than 36°C. This narrowing of the intended fluid temperature difference between the groups may have hindered the detection of a treatment effect and this represents a significant limitation in study design. To improve the current study design, measures should be taken to maintain the pre-warmed fluid temperature using fluid bag insulation or in-line warming devices.

The efficacy of using pre-warmed fluids alone has been questioned due to the heat loss which occurs during administration to the patient.

Intravenous in-line warming devices have been shown to reduce perioperative hypothermia compared to room temperature intravenous fluids in patients also receiving convective heating via a warm air blanket (Smith *et al.*, 1998).

However, other studies have shown that pre-warmed fluid, administered shortly after removal from a warming cabinet, is as efficient at preventing peri-operative hypothermia as that delivered through an in-line warming system (Woolnough *et al.*, 2009; Andrzejowski *et al.*, 2010).

Warming efficacy of the in-line devices may be variable depending on desired fluid flow (Turner *et al.*, 2006).

Specifically designed warming devices for irrigation fluid rates have been shown to effectively maintain warmed fluid temperature during arthroscopy (Pit *et al.*, 1996) and laparoscopy (Moore *et al.*, 1997). Utilisation of such a device would have benefitted the current study by preserving the intended warmed fluid temperature throughout the administration period. The development of peri-operative hypothermia is common and may be attributed to the combination of anaesthetic agent induced thermoregulatory disturbance, exposure of clipped areas of skin to a cool ambient environment (Kurz, 2001) and increased evaporative losses from wounds (Roe, 1971) and the respiratory tract (Bickler and Sessler, 1990). Redistributive heat loss is influenced by the core to periphery temperature gradient (Kurz, 2001) which may be increased by agents such as acepromazine and isoflurane which inhibit protective vasoconstrictive responses. The current study demonstrated an initial significant reduction in temperature in the preparatory phase before active warming was instituted; a finding which is consistent with redistribution of body heat and similar to earlier reports (Franklin *et al.*, 2012).

In a large retrospective study by Redondo *et al*. (2012) the prevalence of slight post-anaesthetic hypothermia (36.50-38.49°C) in dogs undergoing orthopaedic procedures was 48%. If equivalent temperature boundaries were applied to the current study, 94.7% of dogs were slightly hypothermic in the post anaesthetic period.

One dog in the current study was moderately hypothermic (36.49-34.00°C) while 37% were in this temperature range in the retrospective study.

In the current study no dogs were severely hypothermic (<34.00°C) while 3.3% were reported to be so in the study by Redondo *et al*. (2012) where the only form of warming was passive insulation provided by blankets.

The small numbers of dogs with moderate and severe hypothermia in this study may be due to the relatively short procedure length, the use of a thermostatically controlled blanket during surgery (Tan *et al.*, 2004; Fanelli *et al.*, 2009; Röder *et al.*, 2011) and the addition of a HME to the breathing circuit, although the value of HMEs has been questioned (Hofmeister *et al.*, 2011).

The benefit to the patients in employing measures to limit heat loss may have been a limitation of this study, since by limiting heat loss it may be more difficult to detect any effect of warmed irrigation fluids (Hofmeister *et al.*, 2011) A study with an experimental dog population could be devised to test the effects of warmed arthroscopy fluids alone.

Variation in breed was not accounted for which may cause coat thickness and subcutaneous fat to act as confounding factors in terms of heat retention. The study design could have been improved by matching dogs according to hair length and breed to eliminate this confounding factor (Tan *et al.*, 2004).

The temperature of arthroscopy irrigation fluid actually delivered to the patient was not measured in this study and this omission must be considered a weakness in light of the evidence from man highlighting the difficulty in maintain the temperature of such fluids (Kim *et al.*, 2009). The ability to maintain and monitor fluid temperature during administration must be incorporated into the study deign if further work is to be carried out.

Mean delivered isoflurane concentration was not included in data analysis in this study but may potentially affect thermoregulation by virtue of its vasodilatory properties (Kurz, 2001) and it would have been useful to compare the groups to ensure there was no disparity in delivered isoflurane concentrations. Power calculations were based upon a minimal detectable difference of 1°C in line with data reported in man and sample size in the current study was similar to that used in comparable human studies (Kelly *et al.*, 2000; Board and Srinivasan, 2008; Kim *et al.*, 2009).

Whilst we were unable to include the planned number of dogs for this study, we did recruit sufficient dogs to meet our sample size criteria and so the study was sufficiently powered for the predetermined clinically significant effect. If smaller benefits were to be considered clinically significant then a larger sample size would be required to detect them.

The overall significant reduction in body temperature experienced by the dogs in the current study reiterates the requirement for measures to be taken to reduce peri-operative heat loss particularly in the preparatory phase. Whilst in this study there is no detectable benefit to the use of warmed arthroscopy fluids in dogs, the positive results reported in man remain intriguing (Board and Srinivasan, 2008; Kim *et al.*, 2009).

Future studies should examine the effect of warmed irrigation fluids during longer surgeries and incorporate measures to maintain and monitor the temperature of fluids used for arthroscopy during the surgical procedure.

## Conclusion

The results of this study suggest that warmed arthroscopic lavage fluids delivered as described in this paper offer no clinically significant advantage in the maintenance of body temperature.

## Conflict of Interest

The authors declare that they have no conflict of interest.
